# No prognostic value of routine cerebrospinal fluid biomarkers in a population-based cohort of 407 multiple sclerosis patients

**DOI:** 10.1186/s12883-015-0330-4

**Published:** 2015-05-13

**Authors:** Madlyne Becker, Clotilde Latarche, Emilie Roman, Marc Debouverie, Catherine Malaplate-Armand, Francis Guillemin

**Affiliations:** INSERM, CIC-EC, CIC 1433, F-54 000 Nancy, France; Departement of Clinical Epidemiology and Evaluation, Nancy University Hospital, F-54 000 Nancy, France; Université de Lorraine, Université Paris Descartes, EA 4360 Apemac, F-54 000 Nancy, France; Metz-Thionville Hospital, Bel Air Hospital, Departement of Biology, F-57 100 Thionville, France; Department of Neurology, Nancy University Hospital, F-54 000 Nancy, France; Department of Biochemistry and Molecular Biology, Nancy University Hospital, F-54 000 Nancy, France; Department of Clinical Epidemiology and Evaluation, CHU de Nancy, Hôpitaux de Brabois, Allée du Morvan, 54500 Vandoeuvre Les, Nancy, France

**Keywords:** Multiple sclerosis, Cerebrospinal fluid, Prognosis, Disability, Total protein factor, Oligoclonal IgG bands

## Abstract

**Background:**

We aimed to determine the association of clinical and routine cerebrospinal fluid biochemical markers (total protein, IgG index and oligoclonal bands) with disability in multiple sclerosis and whether these biomarkers assessed at diagnosis add prognostic value.

**Methods:**

We followed a cohort of patients included in the Multiple Sclerosis Lorraine Register (eastern France) who had a diagnosis of multiple sclerosis for at least 5 years, as well as biological markers values and MRI findings (Barkhof’s criteria). In a Cox regression model, endpoint was time to score of 4 on the Expanded Disability Status Scale (EDSS) (i.e., limited time walking without aid or rest for more than 500 m).

**Results:**

For 407 patients included, the median time from multiple sclerosis onset to EDSS score 4 was 4.5 years [2.2–7.2]. Cerebrospinal fluid total protein factor < 500 mg/L was associated with EDSS score 4 on bivariate analysis (hazard ratio 0.66, 95% confidence interval 0.46–0.95, p = 0.02). On multivariate analysis, older age at disease onset (≥50 years) and initial primary progressive course of MS but not biological markers predicted worse prognosis.

**Conclusion:**

Routine cerebrospinal fluid biological markers at diagnosis were not prognostic factors of multiple sclerosis progression.

## Background

Analysis of cerebrospinal fluid (CSF) has gained renewed interest in diagnosis of multiple sclerosis (MS) [1,2], but any prognostic value for disease progression is debated. Among CSF markers, oligoclonal IgG bands (OCGBs) are present in more than 95% of patients with MS [3,4] and may have utility and prognostic value for MS diagnosis [5]. Another prognostic CSF marker may be IgG index [6]. Some studies [7,8] have indicated a correlation between index of IgG synthesis or number of OCGBs in CSF and progression rate, but others have not [5,9-12]. Low number or absence of OCGBs in CSF at diagnosis may predict better prognosis, particularly in terms of disability [5,9,10,13-20]. However, the prognostic value has not been established [21].

Among the various prognostic factors of disability in MS, both primary progressive (PP) and remittent relapsing (RR) MS, the number of relapses in the first years and MRI-determined lesional changes seemed to be the most reliable [22-27]. However, most authors agree that the clinical course cannot be predicted by initial symptoms alone [22]. A short interval between the 2 first relapses [26], a rapid early course [27], a primary progressive course without remission [25-27] or onset at older age [26-28] have all been associated with worse long-term prognosis [29]. Sex may influence disease progression (female [23,25]; male [27]). CSF markers with prognostic value at diagnosis would provide the physician with useful information early in the disease course to adjust treatment, without waiting for further clinical manifestations.

We used a population-based MS cohort to determine at MS diagnosis the role of routine CSF biochemical variables, particularly IgG index and presence of OCGBs, on disability (i.e., whether these biomarkers assessed at diagnosis have prognostic value).

## Methods

### Source of the population

Patients were identified in the register of MS patients in the Lorraine region of France. The main purpose of this register is to record all incident cases of MS in Lorraine [23,30] and follow them over time according to neurologists’ routine practice [31,32]. On November 1, 2011, 4,717 patients were registered.

### Patient sampling

**Figure 1:**
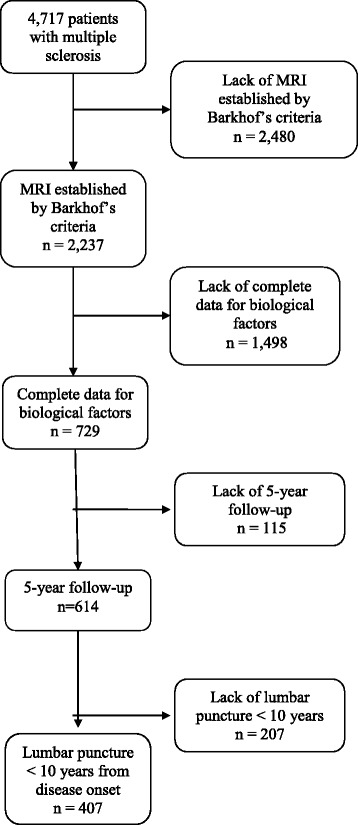
Flow chart of patient selection according to inclusion criteria.

Inclusion criteria were time from MS onset (first signs or symptoms) of at least 5 years [24], a lumbar puncture within 10 years after the MS onset, with complete routine CSF biological data available (CSF OCGBs are stable over time [33-35] thus reflecting status at diagnosis if puncture was performed later), and Barkhof’s criteria available. Only patients with total protein, IgG index and oligoclonal bands available were included in the study. The clinically isolated syndrome (CIS) and radiologically isolated syndrome (RIS) patients were excluded. From the 4,717 patients potentially eligible for the study, 407 patients fulfilled the inclusion criteria (1997-2006) (Figure 1).

The collection of register data was approved by the French Advisory Committee on the Information processing Research in the Field of Health (CCTIRS; reference N°10.258) in May 2010**,** and the French National Commission for Data Protection and Liberties (CNIL; reference N° 909089) in June 2006. Confidentiality and safety of the data were ensured in accordance with these recommendations. An informed consent for use of patient data was obtained from each participant.

### Data collection

For the register, the neurologists collect data at diagnosis and at each following routine consultation. When patients were notified to the register, all information since MS onset was collected retrospectively, and patients were entered in a prospective follow-up.

Data consistency is checked by use of the European Database for MS (EDMUS) software with automatic controls to reduce the frequency of incomplete or incorrect data [36].

For this study, we used demographic data; key episodes in the course of MS, including date of MS onset, date of first and second episodes, and status of recovery from the first episode at the first relapse; Expanded Disability Status Scale (EDSS) score, used to assess MS disability [37], at each visit; and time of assignment of the irreversible disability score, EDSS score 4 (EDSS 4; i.e., limited walking without aid or rest for more than 500 m). MRI findings interpreted by initial Barkhof’s criteria which are: at least one Gadolinium-enhancing lesion or ≥9 T2 lesions, one Juxtacortical lesion, one Infratentorial lesion and three Periventricular lesions; [38] were recorded. As most patients had MRI performed at Nancy University hospital, MRI reading process was consistent for these criteria.

Recovery from the first relapse was classified as incomplete (persistence of neurological signs, corresponding to an EDSS score of at least 2) or complete (absence of neurological signs, an EDSS score of 0 or 1). The onset date of the second neurological episode of MS, which may be a relapse or the onset of the progressive phase, was systematically determined whenever appropriate.

### Biological samples and biochemical analyses

All analyses were performed at the biochemistry laboratory of the University Hospital of Nancy or at the biology laboratory of the Regional Hospital of Thionville. Matched CSF and serum were collected in the clinical setting. All samples were immediately transported at room temperature to the lab. Determination of leukocytes count in CSF was assessed using a Nageotte hematocytometer and microscopic observation. The presence of more than 5 cells per mm3 was considered abnormal. Then, CSF were quickly centrifuged at 2,000 × g for 10 min at 4°C and stored at -20°C. The level of CSF total protein was measured by turbidimetry in both laboratories. Concentrations of albumin and IgG were determined in CSF and in serum by automated turbidimetry or nephelemetry, respectively. These parameters were used to calculate IgG index as (CSF IgG/serum IgG) × (serum albumin/CSF albumin). A comparative analysis showed no significant variation of the IgG index between both methods. The threshold value for IgG index was set at 0.60 (personal unpublished data).

The presence of OCGBs in unconcentrated CSF was determined by isoelectric focusing (IEF) followed by immunoblotting [3]. Sensitivity for detecting OCGBs in CSF using this “gold standard” method is higher than 95%, whatever the commercially available IEF apparatus used [1]. A positive control was systematically used in each gel run to determine the reliability of any given run. Cut-off for OCGBs positivity was defined as 2 CSF-restricted IgG bands. Because the presence of OCGBs in CSF remains constant over the course of the disease, results obtained within 10 years after MS onset, where this technique was used, was considered present at diagnosis [33-35].

### Statistical analysis

Quantitative data are expressed as mean ± SD, median and range, quartiles and extreme values and qualitative data by numbers and percentages. The normality of variables was tested by the Kolmogorov-Smirnov test, skewness and kurtosis. Comparison of 2 groups used chi-square test for categorical variables and Student *t* test for continuous variables. The time from disease onset to assignment of the irreversible score EDSS 4 was considered the endpoint. Subjects without this score and those who died before the first occurrence of an irreversible EDSS score were censored at the last follow-up. The time to the endpoint was described by Kaplan-Meier estimates. Bivariate analyses involved the log-rank test.

The Cox proportional-hazards regression model was used to assess the predictive value of presence of OCGBs and elevated IgG index on disability progression by multivariate analysis, with adjustment for potential confounding variables: age at disease onset, gender, initial form of MS, incomplete recovery at first relapse, protein level, leukocyte count, IgG index, OCGBs, MRI lesions (Barkhof’s criteria), and time from MS onset.

Also, we defined a treatment variable, with treatment (both immunomodulatory or immunosuppressive therapies be it orally or intravenously administered) as present as soon as the first treatment longer than six months before the time to assignment of irreversible EDSS4 for each patient. We considered that this six months time threshold was the minimal time after which we could estimate the beneficial role of the treatment. We introduced the treatment variable in the multivariate analysis.

Stepwise selection of variables was used with p = 0.20 for entering the model and p = 0.10 for staying in the model. We tested the proportionality assumption and the log linearity hypothesis. Because the proportional-hazards assumption was not verified for some covariates, we used a time-varying variable-extended Cox model [39]. Hazard ratios (HRs) and their 95% confidence intervals (95% CIs) for disability progression (time to EDSS 4) were calculated for parameters and were considered significant at *p* < 0.05.

Statistical analysis involved SAS v9.3 for Windows (SAS Inst., Cary, NC, USA).

## Results

Baseline characteristics are in Table 1. The mean age at MS onset was 33.0 ± 11.1 years. The median time between MS onset and CSF testing was 4.6 years [1.0–7.0]. Among registered patients, those included and not included in the study did not differ in age at onset (p = 0.41) and gender (p = 0.31) but did differ in the initial form of MS (p = 0.01) and incomplete recovery at first relapse (p = 0.02).

**Table 1:** **General characteristics of 407 patients with multiple sclerosis (MS)**

	**N**	**%**	**Mean ± SD**
**Age at onset of MS, years**			
<20	59	14.5	
20–28	110	27.0	
29–38	119	29.2	
39–49	87	21.4	
≥50	32	7.9	
**Sex**			
Male	119	29.2	
Female	288	70.8	
**Incomplete recovery from first relapse**			
No	346	85.0	
Yes	61	15.0	
**Initial course of MS**			
Remitting-relapsing	349	85.7	
Primary progressive	58	14.3	
**CSF total protein level, mg/L**			
0–500	312	76.7	
≥500	95	23.3	
**CSF IgG index**			
<0.60	219	53.8	
≥0.60	188	46.2	
**CSF oligoclonal bands**			
No	53	13.0	
Yes	354	87.0	
**Leukocyte count**			
Normal	151	37.1	
Increased (≥5 mm^3^)	256	62.9	
**Barkhof’s criteria (initial MRI)**			
Negative	48	11.8	
Positive	359	88.2	
**Period of MS onset**			
<01/01/98	60	14.7	
≥01/01/98	347	85.3	
**Time from MS onset to: (years)**			
Lumbar puncture	407		4.3 ± 3.2
Last follow-up	407		8.9 ± 3.8

EDSS: Expanded Disability Status Scale; CSF: cerebrospinal fluid; MRI: magnetic resonance imaging.

During the initial course of MS, 349 patients had RR disease at disease onset and 58 PP disease (Table 1). In all, 142 patients had reached EDSS 4. The median time from MS onset to assignment of irreversible EDSS4 by the Kaplan-Meier method was 4.5 years [2.2–7.2]. In total, 188 (46.2%) had elevated CSF IgG index and 354 (87%) CSF OCGBs. The mean IgG index was 0.6 ± 0.7 and mean CSF total protein level 345.5 ± 219.7 mg/L. The proportion of CSF OCGBs was the same whatever the MS course, namely 87.1% and 86.2% of CSF OCGBs positives in RR and PP patients, respectively.

Table 2 gives the results of bivariate analyses of variables associated with median time from MS onset to EDSS4. Among biological variables, CSF total protein level < 500 mg/L was associated with EDSS 4 (HR 0.66, 95% CI 0.46–0.95). Older age at MS onset (≥29 years) was associated with poor prognosis; for age between 29 and 38 years, the HR was 2.56 (95% CI 1.29–5.07) and between 39 and 49 years, 2.58 (1.27–5.25). Univariate analysis showed no significant association of Barkhof criteria (HR 0.8, 95% CI 0.48–1.4; p = 0.4). Other variables associated with good prognosis were the RR form of MS (HR 0.15, 95% CI 0.10–0.21), female sex (HR 0.45, 95% CI 0.32–0.62) and undergoing a treatment (HR 0.20, 95% CI 0.14–0.28).

**Table 2:** **Association of patient characteristics and median time from MS onset to expanded disability status scale score 4**

**Characteristics**	**N**	**Events**	**Median time to EDSS4 (year)**	**p value**
**Age at onset of MS**				
<20	59	10		0.0001
20–28	110	28	19.1	
29–38	119	48	10.0	
39–49	87	32	12.2	
≥50	32	24	4.2	
**Sex**				
Male	119	60	8.0	0.0001
Female	288	82	16.6	
**Initial course of MS**				
Remitting-relapsing	349	92	16.6	0.0001
Primary progressive	58	50	3.8	
**Incomplete recovery from first relapse**				
No	346	117	19.1	0.3
Yes	61	25	11.9	
**CSF total protein level, mg/L**				
0–500	312	100	19.1	0.02
≥500	95	42	10.6	
**CSF IgG index**				
<0.60	219	83	16.6	0.6
≥0.60	188	59	11.4	
**CSF oligoclonal bands**				
Yes	354	119	16.6	0.7
No	53	23	12.2	
**Leukocyte count**				
Normal	151	57	11.9	0.2
Increased (≥5 mm^3^)	256	85	16.6	
**Barkhof’s criteria (initial MRI)**				
Positive	359	129	16.6	0.4
Negative	48	13		
**Treatment**				
Yes	303	228	16.6	0.0001
No	104	37	4.9	

P value: Kaplan-Meier method and Log-Rank Test; EDSS: Expanded Disability Status Scale; CSF: cerebrospinal fluid; MRI: magnetic resonance imaging.

The time to assignment of irreversible EDSS 4 did not differ for patients without and with OCGBs (5.4 vs. 4.9 years, p = 0.52) and for patients with and without IgG index < 0.60 (4.9 vs. 5.2 years, p = 0.65). This did not differ when varying the IgG index threshold to < 0,65 (p = 0,73) or to < 0,7 (p = 0,81).

Table 3 presents the results of multivariate analysis with extended Cox regression models and reveals no significant impact of routine CSF biological markers on assignment of EDSS 4. Even when adjusting for major covariates at onset (age) and at diagnosis (sex, initial course of MS, Barkhof’s criteria, treatment), older age at onset (≥50) (HR 2.48, 95% CI 1.11–5.55) and initial PP course (HR 3.12, 95% CI 2.03–4.79) were associated with poor prognosis, while OCGBs (HR 0.76, 95% CI 0.45–1.30) and IgG index (HR 1.36, 95% CI 0.92–2.02) were not.

**Table 3:** **Prognostic factors at diagnosis associated with EDSS score 4 (n = 407)**

		Multivariate analysis*		
**Characteristics**		**HR**	**95% CI**	**P value**
Age at MS onset (ref age < 20)	20–28	1.25	0.60; 2.57	0.3
	29–38	1.51	0.74; 3.06	0.08
	39–49	1.43	0.68; 2.98	0.1
	≥50	2.48	1.11; 5.55	0.01
Sex (ref men)	Women	1.42	0.99; 2.05	0.1
Initial course of MS (ref primary progressive)	Remitting-relapsing	0.20	0.13; 0.31	0.0001
Incomplete recovery from the first relapse (ref yes)	No	1.10	0.69; 1.75	0.9
CSF total protein level (ref ≥ 500)	≤500 mg/L	0.73	0.49; 1.08	0.4
CSF IgG-index (ref ≥ 0.6)	<0.6	1.36	0.92; 2.02	0.2
CSF oligoclonal bands (ref yes)	No	0.76	0.45; 1.30	0.6
Leukocyte count (ref normal)	≥5 mm^3^	1.08	0.74; 1.56	0.9
MRI Barkhof’s criteria (ref positive)	Negative	1.11	0.25; 4.91	0.9
Treatment (ref no)	Yes	0.29	0.20; 0.42	<.0001

Multivariate analysis*: Extended Cox model; HR: hazard ratio; 95% CI: 95% confidence interval; EDSS: Expanded Disability Status Scale; CSF: cerebrospinal fluid; MRI: magnetic resonance imaging.

## Discussion

We could not demonstrate any value of CSF IgG index and presence of OCGBs at a mean of 8.9 ± 3.8 years of follow-up for prognosis of disability in MS, which contrasts with previous studies [5-12,18] but is in line with a recent study [40]. Older age at disease onset (≥50 years) and initial PP course of MS were predictors of worsening disability. In contrast with other studies [23,25,27,41,42], gender was not a prognostic factor.

In recent years, several studies have suggested the prognostic value of OCGBs in MS, showing good correlation between disability and presence of OCGBs [9,10,16,43], as well as IgG index [44]. CSF OCGBs are stable over time [33-35], which allowed us to consider it for subjects with late lumbar puncture, as a reflection of the value of this factor at diagnosis. However, whether CSF total protein level and IgG index at the time of lumbar puncture actually reflects their values at diagnosis is uncertain.

Many studies have investigated the prognosis of MS in terms of biological markers [21,40,45]. Most studies have focused on the RR form of MS. The absence of OCGBs and low baseline T2-weighted lesions found on MRI were favourable prognostic factors influencing the clinical response to interferon β treatment in patients with RR MS [16]. Age at disease onset and number of MS attacks during the first 2 years of MS were predictors of the evolution of the disability [46]. Blood levels of gene markers [47], CSF IgM oligoclonal bands [48], the association of intrathecal Ig synthesis and cortical lesions [49], simple detection of intrathecal IgG synthesis [9,44] and initial relapse of RR MS have been studied as prognosis markers of RR MS. Other studies have focused on imaging and motor-evoked potentials [50] or the secondary progressive phase of MS [42].

Another recent study found that the presence of OCGBs and elevated total CSF IgG and protein levels moderately were associated with PP MS but not disease progression [40]. As in our study, for patients who had undergone multiple lumbar punctures, only the earliest puncture was considered, and we found the same percentages for the RR form (85.7%) and PP form (14.3%). However, the study differed from ours in a higher number of subjects, different biological technique for CSF analysis, smaller time from MS onset to lumbar puncture and no multivariate analysis.

In patients with clinically isolated syndromes, the presence of lesions as determined by T2-weighted baseline magnetic resonance imaging (MRI) of the brain or medullar tract increases chances of developing multiple sclerosis. The degree of long-term disability from multiple sclerosis correlates with the volume increase of the lesions seen on the brain MRI in the first five years only [11,24] In our study, the change in volume over time was not taken into account. Therefore, the absence of a prognostic role of baseline MRI considering only the Barkhof-Tintore criteria is not surprising.

For an early prognostic perspective, we entered only covariates assessed at the time of MS onset in the regression model. Compared with the other studies, we were interested in the well-known prognostic markers and new data at MS onset.

End of the 90’s was a milestone in the support of MS. As noted earlier, the biological techniques to detect OCGBs have evolved over time, especially after the 1990s, which must be considered in interpreting results. In recent years, the isoelectric focusing technique of CSF with IgG immunoassays was allowed for detecting chronic inflammatory CSF, especially MS. Here, percentage of OCB negative patients is higher (12.9% for RR and 13.8 for PP) than in previous works [10,51] where percentage oscillated between 3-5%, with maximum 10.6% in Siritho’s work in 2009. It was reported a tendency for lower prevalences in CSF from MS patients in countries in southern of Europe compared to MS patients in northern countries. Although not available in our study, information about the origin of our patients might contribute to explain this atypical result [3,13,52]. Finally, the first eligible criterion, minimal time from disease onset for at least 5 years, considerably decreased the number of patients with CSF test results, because in recent years, the database has had more complete data from CSF tests.

The strengths of our study are that first, we conducted multivariate analysis unlike most studies evaluating the predictive value of routine CSF biological data which used only bivariate analysis [4,12,17,43,49] and included a low number of patients (<100) [9,10,43,47], except for two studies [19,40]. Furthermore, only 2 studies claimed they used adjustment for variables [10,16]. Yet, with our relatively large sample size (n = 407), our results did not show the predictive value of these biological markers. Such differences might explain the previous contradictory results. Second, patients were followed for a median of 8.7 years (6.2–11.0), a long period as compared with other studies [10,46,49,50].

Our study contains some limitations. The main difficulty was the number of missing data relating to CSF, which has several explanations. First, some neurologists believe that CSF data are not important in the diagnosis of MS, particularly if the clinical and radiological criteria leave no doubt. On another hand, for some patients living far from major care center, it was not always possible to perform lumbar puncture. Second, the information might be available but not yet complete for some patients, which led to our excluding data for some patients. Therefore, our cohort of 407 patients could have been even larger, with higher statistical power. Third, we had an exhaustive database, the Lorraine MS register, with data for about 4,700 patients. From the overall register population, patients who did and did not meet the eligibility criteria were comparable in demographic characteristics. Regarding the quality of the data, patient records in the register are regularly updated when patients see their neurologist. However, although they were identified in the register, patients retained in the sample were likely more severe since more frequently diagnosed at hospital center, as our team reported in a previous paper [53]. But as a reflection of the recruitment from the register, there was more heterogeneity in clinical profile, in routine biological markers and in treatments.

## Conclusions

In conclusion, our study, involving a large cohort of patients and multivariate analysis, could not demonstrate the value of routine CSF biological markers at MS diagnosis for prognosis of disability progression.
